# Hyperarousal features in the sleep architecture of individuals with and without insomnia

**DOI:** 10.1111/jsr.14256

**Published:** 2024-06-09

**Authors:** Tobias Di Marco, Thomas E. Scammell, Kolia Sadeghi, Alexandre N. Datta, David Little, Nurkurniati Tjiptarto, Ina Djonlagic, Antonio Olivieri, Gary Zammit, Andrew Krystal, Jay Pathmanathan, Jacob Donoghue, Jeffrey Hubbard, Yves Dauvilliers

**Affiliations:** ^1^ Idorsia Pharmaceuticals Ltd Allschwil Switzerland; ^2^ Department of Clinical Research University of Basel Basel Switzerland; ^3^ Department of Neurology Beth Israel Deaconess Medical Center Boston Massachusetts USA; ^4^ Beacon Biosignals, Inc. Boston Massachusetts USA; ^5^ University Children's Hospital Basel Basel Switzerland; ^6^ Clinilabs Drug Development Corporation New York New York USA; ^7^ University of California San Francisco California USA; ^8^ Centre National de Référence Narcolepsie, Unité du Sommeil, CHU Montpellier, Hôpital Gui–de–Chauliac Université de Montpellier, INSERM INM Montpellier France

**Keywords:** hyperarousal, insomnia, sleep, sleep architecture

## Abstract

Sleep architecture encodes relevant information on the structure of sleep and has been used to assess hyperarousal in insomnia. This study investigated whether polysomnography‐derived sleep architecture displays signs of hyperarousal in individuals with insomnia compared with individuals without insomnia. Data from Phase 3 clinical trials, private clinics and a cohort study were analysed. A comprehensive set of sleep architecture features previously associated with hyperarousal were retrospectively analysed focusing on sleep–wake transition probabilities, electroencephalographic spectra and sleep spindles, and enriched with a novel machine learning algorithm called the Wake Electroencephalographic Similarity Index. This analysis included 1710 individuals with insomnia and 1455 individuals without insomnia. Results indicate that individuals with insomnia had a higher likelihood of waking from all sleep stages, and showed increased relative alpha during Wake and N1 sleep and increased theta power during Wake when compared with individuals without insomnia. Relative delta power was decreased and Wake Electroencephalographic Similarity Index scores were elevated across all sleep stages except N3, suggesting more wake‐like activity during these stages in individuals with insomnia. Additionally, sleep spindle density was decreased, and spindle dispersion was increased in individuals with insomnia. These findings suggest that insomnia is characterized by a dysfunction in sleep quality with a continuous hyperarousal, evidenced by changes in sleep–wake architecture.

## 1 INTRODUCTION

Chronic insomnia disorder, the most prevalent sleep disorder, affects up to 10% of the adult population globally (Morin & Jarrin, 2022), and is characterized by difficulty initiating and/or maintaining sleep coupled with distress or impairment in daytime functioning. (American Psychiatric Association, 2013). It is a heterogenous disorder influenced by predisposing factors that include personality traits, genetic and epigenetic, and neurobiological factors (Riemann et al., 2022), precipitated by short‐term stressors (Dressle & Riemann, 2023; Spielman et al., 1987), and further perpetuated by factors such as heightened arousal states including unstable rapid eye movement (REM) sleep (Riemann et al., 2012) that contribute to the onset and chronic progression of insomnia (Spielman et al., 1987). The concept of chronic insomnia disorder being a condition of hyperarousal has attracted substantial scientific attention in recent years (Dressle & Riemann, 2023). Multiple lines of evidence support the role of hyperarousal in the pathophysiology of chronic insomnia disorder, including studies showing both an elevated sympathetic activity (Kim et al., 2022) and hypermetabolism in the hypothalamus and the relevant efferent projections of arousal networks, including excessive cortical activity during sleep, in individuals with insomnia (Nofzinger et al., 2004).

Polysomnography (PSG) provides an opportunity to gain objective insights into the characteristics of hyperarousal in chronic insomnia disorder, by comparing individuals with and without insomnia. Analysis of sleep macrostructure suggests that there are marginal differences between individuals with insomnia and those without, including only a 2% reduction in non‐REM (NREM) Stage 3 (N3) and REM sleep (Baglioni et al., 2014). Furthermore, insomnia can be characterized by increased episodes of wakefulness during sleep, as well as an increased vulnerability of NREM stage 2 (N2) sleep indicated by more N2 to N1 sleep‐stage transitions (Andrillon et al., 2020; Wei et al., 2017). While useful, quantitative measures of sleep and wake behaviour derived from standardized scoring methodology do not consistently identify subjects with insomnia and offer less granularity than electroencephalogram (EEG) measures, including spectral analyses or quantification of sleep microarchitecture (e.g. spindle‐derived metrics). An increasing body of research has focused on these aspects of EEG, particularly increased high‐frequency brain activity and a shift towards wakefulness in patients with insomnia as compared with individuals without insomnia (Kay et al., 2016; Levenson et al., 2015; Riemann et al., 2010). Small studies have reported increased alpha and beta EEG activity during Wake and NREM sleep (Shi et al., 2022; Zhao et al., 2021), although this is not consistent in the literature. These discrepancies might be due to the specific subtype of insomnia being examined or the influence of major medical conditions (Cervena et al., 2014; Wu et al., 2013). Furthermore, the potential confounding influence of hypnotic medications with well‐characterized effects on sleep architecture, such as benzodiazepines, can confound the interpretation of existing research findings on the EEG features that characterize patients with insomnia (Kang et al., 2022; Poyares et al., 2004). Inconsistencies extend to other parameters as well, such as changes in the incidence and morphology of sleep spindles – oscillatory bursts of neural activity between 11 and 15 Hz generated by the thalamic reticular nucleus during N2 sleep, believed to aid sleep stability (Fernandez & Lüthi, 2020). The role of spindle generation in insomnia remains uncertain, with studies showing mixed results (Andrillon et al., 2020; Bastien et al., 2009). Generally, these inconsistencies observed in the available literature could be related to factors, such as the heterogeneity of insomnia disorder itself (Buysse et al., 2010) or low sample sizes in many studies (Baglioni et al., 2014).

The hypothesis of this study is that patients with insomnia exhibit quantifiable PSG features indicative of hyperarousal. To enhance the generalizability of current findings, we performed a comprehensive analysis using pooled data from 1710 individuals with and 1455 without insomnia. Our aim was to investigate whether differences in sleep‐stage transitions, spectral features and spindle characteristics exist between individuals with insomnia and those without. This analysis is intended to further explore and potentially support the hyperarousal model of insomnia.

## 2 METHODS

### 2.1 Individuals with insomnia

**FIGURE 1:**
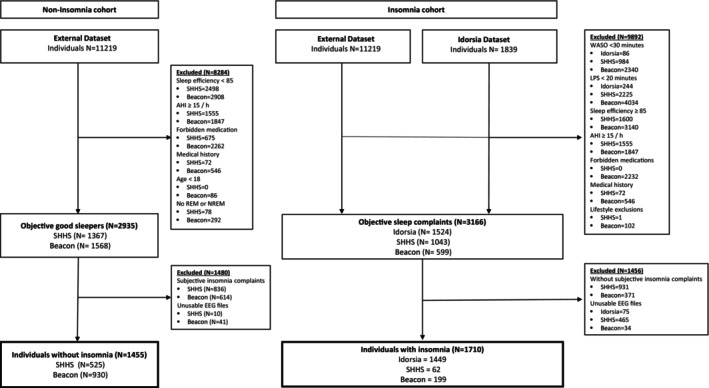
Flow chart for individuals with and without insomnia.

We analysed PSGs from three independent databases: (1) Idorsia Pharmaceuticals Ltd; (2) Beacon Clinico‐PSG Database (Beacon); and (3) the Sleep Heart Health Study (SHHS). The datasets were pooled to enhance the generalizability of our findings and to provide a more comprehensive analysis by integrating diverse data sources. All studies were conducted in accordance with the Declaration of Helsinki, the International Conference on Harmonization Guideline for Good Clinical Practice, and local regulations. All individuals provided written informed consent. Figure 1 presents a detailed flow chart illustrating the various datasets and their numbers in this study.

#### 2.1.1 Idorsia Pharmaceuticals Ltd dataset

This dataset included all individuals who were randomized in the two pivotal Phase 3 studies assessing the efficacy and safety of daridorexant (clinicaltrials.gov: NCT03545191 and NCT03575104; Mignot et al., 2022). For the purposes of this study, only the initial screening PSG was considered, when individuals had not received any study treatment. All individuals were aged 18 years or older, and had a clinical diagnosis of insomnia disorder according to the Diagnostic and Statistical Manual of Mental Disorders 5th edition (DSM‐5) criteria (American Psychiatric Association, 2013), an Insomnia Severity Index© score ≥ 15 (Morin et al., 2011) and a self‐reported history of disturbed sleep (≥ 30 min to fall asleep, ≥ 30 min awake during sleep time, and self‐reported total sleep time [TST] of ≤ 6.5 hr) for more than 3 nights per week for at least 3 months prior to screening. No individuals had an apnea–hypopnea index (AHI) ≥ 15 per hr or an event associated with a blood oxygen saturation level (SpO_2_) < 80% during the screening PSG. Furthermore, individuals with major comorbidities such as acute or unstable psychiatric disorders, alcohol or substance use disorder, periodic limb movement disorder, restless legs syndrome, circadian rhythm disorder, REM behaviour disorder or narcolepsy, and central nervous system active medications (including hypnotics and anxiolytics) not discontinued at least 2 weeks prior to the first study visit were excluded. Full details of the study design and eligibility criteria of these two studies are published elsewhere (Mignot et al., 2022).

#### 2.1.2 Beacon dataset

Individuals with insomnia who received care at a single USA‐based academic medical centre sleep laboratory between 2008 and 2017 were selected from the Beacon Clinico‐PSG Database (Abou Jaoude et al., 2020; Sun et al., 2019). Individuals were considered to have insomnia complaints if they checked at least one of the following: “I have trouble falling asleep”; “I have trouble staying asleep”; “My sleep is being evaluated because of my sleepiness”; “My sleep is being evaluated because of my insomnia”; “I am tired all the time no matter how much sleep I get”; “I typically fall asleep after more than 60 min”; “I typically wake up more than three times a night”; “Poor sleep impacts my day” either “a little”, “somewhat”, “a lot”, or marked sleepiness, fatigue or both when asked: “If ‘sleepiness’ means actually dozing off, while ‘fatigue’ means lack of energy but NOT dozing off, which best describes your symptoms?”. Although some of the questions may be associated with other conditions as well such as narcolepsy or obstructive sleep apnea, these individuals were excluded based on International Classification of Diseases (ICD)‐10 codes (Supplement 1). Individuals who had not filled out a pre‐sleep questionnaire were considered to have insomnia complaints if they had ICD‐10 codes corresponding to diagnoses containing the word “insomnia” in their medical record.

Individuals from the Beacon dataset were excluded if they had an AHI >15 per hr or ICD‐10 codes for psychiatric disorders or neurological conditions within 1 year of the PSG (Supplement 1). Furthermore, individuals were excluded if they were taking sedating antihistamines, centrally acting anticholinergics, stimulants, antidepressants, antipsychotics, anxiolytics, hypnotics, cholinesterase inhibitors, mood stabilizers, anti‐Parkinsonian therapies or anticonvulsants, as these medications may influence sleep or relate to conditions with abnormal sleep, as indicated in their pre‐sleep questionnaire responses, or if a pre‐sleep questionnaire was not present for the PSG encounter, as indicated in their medical record within 1 year of the PSG encounter. All medications leading to exclusion are shown in Supplement 2.

#### 2.1.3 SHHS dataset

Individuals with insomnia complaints were selected from the SHHS (ClinicalTrials.gov: NCT00005275; Quan et al., 1997), a publicly available dataset from a multicentre, community‐based prospective cohort study designed to evaluate associations between sleep‐disordered breathing and cardiovascular diseases. Eligible individuals were at least 40 years old and were recruited from nine existing parent epidemiological studies where data on cardiovascular risk factors had been collected previously. Individuals were considered to have insomnia if they reported at least 16–30 times per month: “Have trouble falling asleep”; “Wake up during the night and have difficulty getting back to sleep”; or “Wake up too early in the morning and be unable to get back to sleep”. For the current analysis, all individuals considered to have insomnia who had successfully completed a baseline PSG were included. In contrast to the Idorsia and Beacon datasets, the SHHS included home PSGs obtained between 1995–1998 and 2001–2003. Full details of the SHHS design have been previously reported (Quan et al., 1997).

Individuals from the SHHS dataset were excluded if they had an AHI >15 per hr, relevant psychiatric disorders or neurological conditions (a medical diagnosis for sleep apnea, restless legs syndrome or narcolepsy), or if they were taking acetylcholine esterase inhibitors for Alzheimer's disease, medications used to treat Parkinson's disease, non‐tricyclic antidepressants other than monoamine oxidase inhibitors, tricyclic antidepressants, benzodiazepines, anticholinergic medications with beta2 antagonists, or within 2 weeks of the recording: oral steroids, oral or inhaled sympathomimetic medications, or weight loss medications, as indicated in SHHS questionnaire responses.

Individuals with insomnia from all three datasets were pooled to create a single insomnia group. All individuals met the following objective PSG criteria: ≥ 30 min wake after sleep onset (WASO); ≥ 20 min latency to persistent sleep (LPS), defined as time from lights off to the first epoch of 20 consecutive epochs of non‐wake (stage N1, N2, N3 or stage REM); and a sleep efficiency < 85% based on PSG evaluation. These criteria were used to mimic the treated population from the daridorexant clinical program (Mignot et al., 2022).

### 2.2 Individuals without insomnia

Individuals without insomnia were selected from the Beacon and SHHS datasets. All individuals met the following conditions: absence of psychiatric disorders or neurological conditions (including insomnia) using ICD‐10 codes (World Health Organization(WHO), 1993); AHI < 15 per hr; and absence of medications belonging to sedating antihistamines, centrally acting anticholinergics, stimulants, antidepressants, antipsychotics, anxiolytics, hypnotics, cholinesterase inhibitors, mood stabilizers, anti‐Parkinsonian therapies or anticonvulsants. Furthermore, individuals without insomnia had a sleep efficiency of ≥ 85% during the PSG night; a sleep efficiency of ≥ 85% is usually associated with adequate sleep quality (Buysse et al., 1989; Miyata et al., 2013). The flow chart indicates the population and the exclusion criteria (Figure 1).

### 2.3 PSG recordings

Across all PSG databases, sleep–wake stages (Wake, N1, N2, N3 and REM) were scored in 30‐s epochs according to the American Academy of Sleep Medicine (AASM) guidelines (Berry et al., 2015). The Idorsia Pharmaceuticals Ltd dataset was collected using the methods of (and analysed by) Clinilabs Drug Development Corporation. Prior to computing any of the features below, EEG signals were re‐referenced using the contralateral mastoid process (M1 or M2), resampled to 200 Hz using a polyphase low‐pass finite impulse response filter computed from a Kaiser window, and high‐pass filtered to 0.5 Hz to correct for variable pre‐filtering settings that were observed across the different datasets. Recordings with a sampling rate less than 200 Hz were up‐sampled (i.e. no frequencies above the Nyquist frequency of the original sample rate would be present). All downstream analyses further band‐passed signals to a range below the Nyquist frequency (50 Hz) of the lowest sample rates (100 Hz). For this analysis, only central electrodes (C3 and C4) were used to align with the SHHS dataset. Adjustments such as resampling EEG signals and focusing on central electrodes were crucial in harmonizing data across different datasets, ensuring the pooled data were as consistent and comparable as possible. A number of individual EEG recordings (625) were deemed unusable for purposes of this analysis for a variety of reasons (e.g. incorrectly labelled channels, duplicate channels, of corrupt signal data; Figure 1).

### 2.4 Sleep–wake transition

To analyse the dynamics of sleep stages during the period between lights off and lights on, the likelihood of transitioning between the five stages, including Wake, was evaluated. Specifically, we counted the number of transitions from one sleep stage in one epoch to another sleep stage (including staying in the same annotated sleep stage) in the next epoch, producing a 5‐by‐5 matrix of counts for each recording. These counts were used to estimate the probabilities of transitioning to each of the five sleep stages in the next epoch given the current epoch's sleep stage; see the Statistical Modelling subsection below.

### 2.5 Spectral analysis

Band powers were estimated using multi‐taper spectral density estimation (Thomson, 1982). Multi‐taper estimation leverages multiple, orthogonal measurements (multi‐taper windows) for each spectral bin to reduce bias and variance in spectral power estimates. Spectral features were derived using the power in four frequency bands (*δ*: 0.5–4 Hz; *θ*: 4–8 Hz; *α*: 8–12 Hz; *β*: 12–30 Hz; Chikhi et al., 2022), computing an average for each band across all epochs of a given sleep–wake stage (five stages in total), summing up to a total of 20 features. First, artefactual regions of the EEG were rejected: the root‐mean squared (RMS) amplitude was calculated for each 3‐s segment of the recording, and segments with an RMS amplitude ≤ 1 μV or ≥ 250 μV were considered artefacts and excluded from downstream analyses. These thresholds were selected to be relatively conservative as they fall well outside the physiological range of approximately 10–100 μV (Ernst Niedermeyer & Da Silva, 2005). The median fraction of a recording rejected as artefactual was 0.0013. No full recordings were rejected due to artefacts. No electrooculogram correction was applied as only the central electrodes were used: note that we included only two central lead channels across all analyses, as these were the only electrodes available in the SHHS dataset. After artefact rejection, spectral power was computed from 2‐s multi‐taper windows with 1‐s overlap. If a spectral window overlapped with a window marked as artefactual, the spectral window was not included in the analysis.

The four band powers in the 2‐s windows were averaged to 30‐s windows and summed across lateral channels C3 and C4. Given the high variance of absolute power found across recording sites, analyses were focused on the four relative power bands: for each of the four absolute power bands (*δ*, *θ*, *α*, *β*), the relative power was computed by dividing the power in each band by the sum of the power of all four bands within the same 30‐s window and channel. The 30‐s windows were aggregated to the sleep stage by taking the mean across all epochs of each stage (i.e. N1, N2, N3, REM and Wake) in each recording. These spectral features were then log‐transformed to reduce skew.

### 2.6 Spindle analysis

Spindle features were calculated using the open‐source Luna package (version 0.23; https://zzz.bwh.harvard.edu/luna/ref/) following the methods from Purcell et al. (Ernst Niedermeyer & Da Silva, 2005). To maintain consistency with the use of the Luna spindle analysis, artefact rejection for spindle analysis followed the conventions described in the Luna package. Briefly, we resampled signals to 128 Hz, low‐pass filtered them at 35 Hz, and removed artefacts by computing power within the delta band, rejecting epochs that had more than 2.5 times the average delta power in a 15‐epoch sliding window.

After artefact removal, spindles were detected within all N2 stages by first convolving a Morlet wavelet (13.5 Hz) over the signal and then smoothing the convolution's magnitude using a sliding window of 0.1 s. Spindles were detected from this convolution by thresholding: at least 0.3 s had to be over 4.5 times the mean of all N2 epochs, and in a 0.5‐s window around this region, power had to be at least twice this N2 epoch mean. These putative spindles were merged if they fell within 0.5 s of one another, and any that lasted longer than 3 s were rejected. This basic approach to spindle validation, via Morlet wavelets, has been validated against manual spindle annotation (Younes et al., 2015).

Because sleep spindles are known to couple with slow oscillations (SOs) during N2 sleep, we detected SOs by: (1) low‐pass filtering the entire signal at 4.5 Hz (note that the signal was already high‐pass filtered at 0.5 Hz to reduce site‐to‐site variability); and (2) within the N2 stage, marking all consecutive positive‐to‐negative zero‐crossings that fall between 0.8 and 2 s in length as a SO.

Having detected both spindles and slow waves, we computed the following four features (spindle density, spindle dispersion, SO phase at spindle peak for fast and slow spindles) per channel (C3 and C4), for a total of eight unique features. Note that, because the underlying data are discrete events and can be spatially sparse, not all of these features can be sensibly averaged across channels; this is why spindle features were kept separate per channel. Two of the features were computed for all spindles (total range of 11–15 Hz): density (spindle count in 1 min) and dispersion. We calculated spindle dispersion (how variable spindle counts are across 30‐s epochs) by dividing the variance of the spindle counts across epochs of N2 by the average number of spindles across 30‐s epochs of N2. The remaining four features, i.e. the SO spindle phase peak (SO phase at spindle peak) were calculated for fast (≥ 13–15 Hz) and slow (11 – < 13 Hz) spindles for each channel. The SO phase at the spindle peak was determined by inspecting each detected spindle that occurred during a detected SO. We compared the peak of the spindle to the start and end of the SO, reporting when the peak occurred relative to these two positions as an angle between 0 and 360 degrees. The start of the SO was defined as the preceding zero‐crossing from positive to negative (relative to the spindle start), and the end was the subsequent such zero‐crossing.

### 2.7 Wake EEG Similarity Index (WESI)

We developed the WESI base, in part, on an odds‐ratio product calculation (Younes et al., 2015). The objective was to provide a continuum of sleep/wake state that is robust to the mixed datasets and populations considered here. To generate the WESI model, we used training data from a random sample of 80% of the data from all three of our datasets (Idorsia, Beacon and SHHS) including both groups: insomnia and non‐insomnia individuals. We trained WESI by labelling all segments that occurred during a sleep stage (i.e. N1, N2, N3 or REM) as 0 and all segments that occurred during a Wake period as 1. Spectral power in the delta, theta, alpha and beta frequency bands was computed for every 3‐s window and transformed into model features in the following steps: (1) divided by the sum of powers *δ* + *θ* + *α* + *β* to obtain the relative power in each band; (2) logit‐transformed the relative powers: 𝑙𝑜𝑔(𝑥/(1 − 𝑥)); (3) computed the *z*‐score for each WESI feature as estimated by the training set data; (4) computed all interaction terms (e.g. $\text{alpha power}\;x\;\text{beta power},{\text{alpha power}}^{2}$) for a total of 14 features (four power bands, six power–power interactions, and four squared power bands). The 15 regression parameters (one per feature, plus an intercept) were fit to these labels using a L1‐regularized logistic regression (𝜆 = 1𝑒 − 1) with a Huber‐loss function (𝛿 = 3). This regularization and robust loss function was employed to help ensure our model yielded reasonable out‐of‐sample performance.

The WESI was then validated on the test data, which comprised the remaining 20% of data excluded from training to ensure it accurately identified out‐of‐sample segments as being from wake or sleep periods, thus mitigating the risk of overfitting. The purpose of validating the model on the test data was to ensure that the model correctly learned a function that separates most of the wake periods from most of the sleep periods, and that the accuracy was similar across the datasets. With such short segments it was to be expected that some sleep‐labelled segments appear more like a period of wakefulness. We evaluated the model using its classification accuracy, and the AUC (the area under the receiver operating curve, ROC). The AUC provides a summary of model performance independent of the trade‐off between model hit rate and precision: specifically, the ROC gives the false‐positive rate of the model for any given hit rate. WESI correctly labelled 78.6% (0.86 AUC) of the 3‐s segments of the test data for Idorsia, 78.5% (0.85 AUC) for SHHS, and 79.8% (0.86 AUC) for Beacon.

In our results we report the mean WESI values for each sleep stage (a total of five features). The statistical analysis of these values was performed on a logit‐transformed scale, to ensure statistical modelling assumptions were satisfied, and we report model coefficients and confidence intervals along a linear scale. To compute the linear‐scale means and confidence intervals from the logit‐scale model, we used a finite difference approximation (validated by bootstrapping 10,000 samples from the population generating the most extreme mean, and demonstrating a difference of less than 0.001).

### 2.8 Statistical analysis

Statements around statistical significance were determined using linear mixed‐effects regression models for spectral, spindle and WESI feature types. All models included terms for age and sex, correcting for the potential impact of these factors on EEG features when making comparisons across groups.

For sleep‐stage transition counts, a large number of recordings did not contain some of the transitions. When necessary, we used a Hurdle model (see below for further discussion of this model design) when at least 1.5% of recordings contained the transition (transitions that occurred in fewer than 1.5% of recordings were not modelled at all) and no more than 98.5% of the recordings contained the transition, otherwise we used a Generalized Mixed Effects model.

For all models, the covariates for the regression were: age (centred at 50 years old), sex and group (insomnia and non‐insomnia individuals). In addition to age and sex, a random effect for testing site was also included in the model to account for potential variation introduced by where the recording took place. The Idorsia dataset included a total of 109 sites with standardized recordings, and the Beacon and SHHS datasets included one site each. A null, main effect and full interaction model were used, with the following formulas.

Null model:

$$\text{feature}=1+\mathrm{age}*\mathrm{sex}+\left(1|\text{study}\_\text{subject}\_\mathrm{id}\right)+\left(1|\text{site}\right).$$

Main effect model:

$$\text{feature}=1+\mathrm{age}*\mathrm{sex}+\text{group}+\left(1|\text{study}\_\text{subject}\_\mathrm{id}\right)+\left(1|\text{site}\right).$$

Interaction model:

$$\text{feature}=1+\mathrm{age}*\mathrm{sex}*\text{group}+\left(1|\text{study}\_\text{subject}\_\mathrm{id}\right)+\left(1|\text{site}\right).$$

Features were determined to be relevant if the likelihood ratio test between a null model and the main effect model had an adjusted *p*‐value < 0.05, and an interaction if the likelihood ratio test between the full model and the main effect had an adjusted *p*‐value < 0.05, where *p*‐values were adjusted for multiple comparisons within each type of feature (spectral, spindle, WESI or sleep‐stage transition) using the False Discovery Rate (Hochberg & Y., 1995). Note that the multiplicity comparisons used a larger *N* than the models reported here because initial exploratory work included a larger set of features. Keeping the larger *N* when correcting for multiplicity is the more conservative, and inferentially sound approach. We report the following number of features per type: 20 spectral features (from a total of 180), eight spindle features (from a total of 14), five WESI features (from a total of 10) and 25 sleep‐stage transition features (from a total of 25).

In the case of the sleep‐stage transition analyses, a hurdle model was employed for transitions that occur at least once in 1.5%–98.5% of recordings. In a hurdle model there are two stages: an initial model that predicts the probability that a given transition is observed zero times in a recording (the zero‐count model), and a second model, that is conditioned to predict non‐zero values (the non‐zero‐count model). In the present case, both of these models employed a logistic function as the link in a generalized mixed‐effect linear regression: the zero‐count modelled a single observation (zero versus non‐zero) and the second modelled all counts for the non‐zero observations. Both stages of the hurdle model employed the same covariate structure as used above for the other features. For all sleep‐stage transitions, in addition to requiring significance of the adjusted *p*‐value of the likelihood ratio test between main effect and null models, at least one insomnia coefficient was required to be significant for the transition count to be deemed significantly different between insomnia and non‐insomnia groups.

## 3 RESULTS

### 3.1 Demographic characteristics

A total of 1710 individuals with insomnia from the three datasets were analysed: 1449 individuals from the Idorsia dataset, 199 from the Beacon dataset, and 62 from the SHHS dataset. A total of 1455 individuals without insomnia were included (Beacon, *n* = 930; SHHS, *n* = 525; Figure 1).

The mean age was higher in the insomnia group (insomnia: 56.3 years [standard deviation (SD) 14.8]; non‐insomnia: 50.5 years [SD 14.0]; Table 1). The insomnia group included more females (66%) than the non‐insomnia group (50%), consistent with sex differences reported in insomnia populations.

**TABLE 1:** Demographics, baseline characteristics and sleep parameters of individuals with and without insomnia.

Variables	Insomnia *N* = 1710			Non‐insomnia *N* = 1455		Statistical comparison
	Idorsia	Beacon	SHHS	Beacon	SHHS	
Number of individuals	1449	199	62	930	525	
Demographics and baseline characteristics						
Age, years	56.1 (14.5)	53.8 (15.7)	69.1 (10.7)	45.8 (13.6)	58.7 (10.8)	
	56.3 (14.8)			50.5 (14.0)		*p* < 0.001
Sex %, female	67.8	51.7	70.3	46.0	57.4	
	66.0			50.2		*p* < 0.001
Sleep variables						
Sleep efficiency, %	62 (15)	67 (16)	70 (12)	92 (4)	90 (3)	
	63 (15)			92 (4)		*p* < 0.001
TST, min	296.6 (74.9)	300.9 (80.0)	336.4 (77.1)	410.5 (40.2)	394.3 (52.7)	
	298.6 (75.9)			404.5 (45.8)		*p* < 0.001
LPS, min	79.7 (55.7)	56.0 (48.0)	56.5 (33.1)	10.6 (12.0)	12.9 (12.2)	
	76.0 (54.8)			11.4 (12.1)		*p* < 0.001
WASO, min	112.5 (53.6)	92.7 (52.8)	82.0 (48.9)	22.6 (15.4)	28.3 (13.7)	
	109.0 (53.9)			24.7 (15.0)		*p* < 0.001
N1 sleep, min	39.0 (21.5)	57.9 (37.0)	18.4 (12.9)	47.4 (30.8)	16.1 (10.5)	
	35.9 (24.8)			45.9 (29.5)		*p* = 0.043
N2 sleep, min	168.7 (52.7)	149.5 (55.5)	185.4 (53.2)	217.8 (48.6)	213.5 (49.6)	
	167.1 (53.5)			216.2 (49.0)		*p* < 0.001
N3 sleep, min	36.2 (29.3)	49.5 (35.5)	64.5 (44.7)	71.4 (41.0)	77.5 (41.3)	
	38.8 (31.5)			73.6 (41.2)		*p* < 0.001
REM sleep, min	52.6 (25.1)	44.1 (31.2)	68.0 (27.6)	73.9 (29.5)	87.3 (25.6)	
	52.2 (26.3)			78.8 (28.9)		*p* < 0.001
Awake, min	181.7 (73.2)	141.9 (70.3)	131.7 (59.3)	30.9 (17.4)	38.0 (15.7)	
	175.1 (74.0)			33.5 (17.1)		*p* < 0.001
N1 sleep, %	8.1 (4.5)	13.0 (8.0)	3.9 (2.6)	10.6 (6.8)	3.7 (2.3)	
	8.5 (5.3)			8.1 (6.5)		*p* < 0.001
N2 sleep, %	35.1 (11)	33.6 (11.7)	39.9 (11.2	49.3 (10.1)	49.2 (9.3)	
	35.1 (11.1)			49.3 (9.8)		*p* < 0.001
N3 sleep, %	7.5 (6.1)	11.1 (7.9)	13.6 (8.8)	16.2 (9.3)	18.1 (9.7)	
	8.2 (6.6)			16.9 (9.5)		*p* < 0.001
REM sleep, %	11.0 (5.2)	9.8 (6.8)	14.4 (5.5)	16.7 (6.5)	20.1 (5.2)	
	10.9 (5.5)			18 (6.2)		*p* < 0.001
Awake, %	37.8 (15.2)	32.2 (16)	28.2 (12)	7 (3.9)	8.7 (3.3)	
	36.8 (15.4)			7.6 (3.7)		*p* < 0.001

*Note*: Data are shown as mean (standard deviation) across datasets. A comparison of each measure across insomnia and non‐insomnia groups is provided using a Chi‐squared test for Sex and Welch's *t*‐test for the other measures.

Abbreviations: Awake, total time awake, from light‐off to lights‐on; Beacon, Beacon Clinico‐PSG Dataset; LPS, latency to persistent sleep; REM, rapid eye movement; SHHS, Sleep Heart Health Study; TST, total sleep time; WASO, wake after sleep onset, defined as wake time after onset of persistent sleep until lights‐on.

Sleep parameter values in individuals with insomnia varied across the three datasets, as detailed in Table 1. The proportions of N2, N3 and REM sleep were lower, and the proportion of time spent awake was higher in the insomnia group compared with the non‐insomnia controls. The proportion of N1 was similar between groups. While statistical tests comparing differences across populations are provided in Table 1 for reference, the pre‐selection of the population infers an inherent bias that makes it difficult to interpret these *p*‐values (Sassenhagen & Alday, 2016).

### 3.2 Comparison of sleep–wake transitions probabilities in individuals with and without insomnia

**FIGURE 2:**
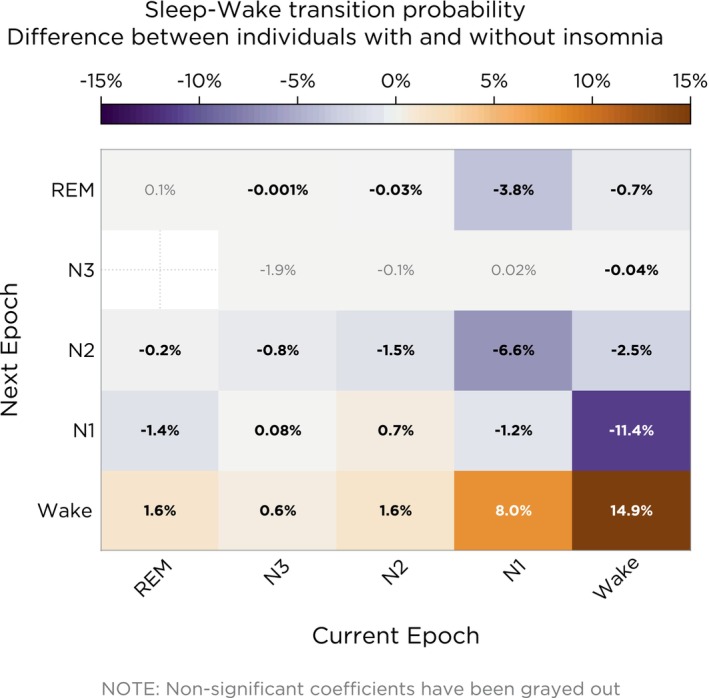
Transition matrix illustrating the model‐estimated probabilities of sleep–wake transitions. The colour gradient represents the percentage change in transitions for individuals with insomnia as compared with those without insomnia. Bold entries represent transitions with a statistically significant difference (*p* < 0.05).

Individuals with insomnia, as compared with controls, had a higher probability to transition to Wake from every stage (range: 0.6%–14.9%, *p* < 0.05) and lower probability to transition from Wake to all sleep stages (range: −0.04% to −11.4%, *p* < 0.05; Figure 2). N2‐to‐Wake and N2‐to‐N1 transitions were increased (1.6% and 0.7%, respectively, *p* < 0.05), whereas transitions from N2‐to‐N2 were decreased (−1.5%, *p* < 0.05) in individuals with insomnia compared with controls. Additionally, transitions from N1‐to‐N1, N1‐to‐N2 and N1‐to‐REM were decreased (*p* < 0.05) in individuals with insomnia compared with controls. Finally, individuals with insomnia had fewer transitions from Wake‐to‐REM and from N1‐to‐REM (−0.7% and −3.8%, respectively, *p* < 0.05) compared with those without insomnia. The absolute transition probabilities per group are reported in Figure S3.

### 3.3 Comparison of relative spectral features in individuals with and without insomnia

**FIGURE 3:**
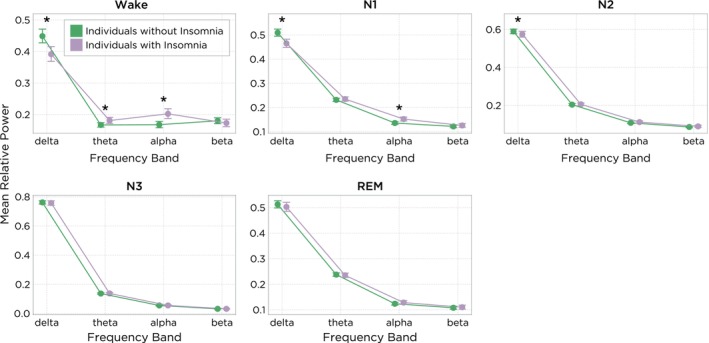
Relative spectral features in individuals with and without insomnia (mean, 95% confidence interval [CI]) within each sleep stage. The *y*‐axis represents the proportion of total power for each frequency band, with a value of 1 corresponding to 100%. *Indicates statistically significant difference (*p* < 0.05) between groups (with versus without insomnia).

Figure 3 shows the full set of spectral features per band, sleep stage and group.

During epochs scored as Wake, the mean relative alpha power was 20.25% (95% confidence interval [CI]: 18.76%, 21.86%) and 16.81% (95% CI: 15.83%, 17.84%), and the mean relative theta power was 18.1% (95% CI: 17.2%, 19.1%) and 16.7% (95% CI: 16.0%, 17.4%) in insomnia and non‐insomnia individuals, respectively. Alpha power was increased by 3.45% (95% CI: 2.49%, 4.49%; *p* < 0.001) in individuals with insomnia. They also had theta power that was increased by 1.40% (95% CI: 0.79%, 2.04%; *p* < 0.001) compared with the non‐insomnia group. The mean relative delta power during Wake was 39.15% (95% CI: 36.88%, 41.56%) and 44.87% (95% CI: 42.75%, 47.10%) in insomnia and non‐insomnia individuals, respectively. The difference in delta power during Wake between these groups was reduced by −5.71% (95% CI: −7.09%, −4.29%; *p* < 0.001) in the insomnia group.

During N1 sleep, similar results were observed as in Wake. In individuals with insomnia, relative alpha power was increased by 1.64% (95% CI: 1.15%, 2.16%; *p* < 0.001) and mean relative delta power was reduced by −4.41% (95% CI: −5.45%, −3.34; *p* < 0.001) as compared with non‐insomnia controls.

During N2 sleep, mean relative delta power was decreased by −1.47% (95% CI: −2.43, −0.48%; *p* = 0.009) when compared with individuals without insomnia. No significant (*p* > 0.05) differences were observed in N3 and REM sleep in the mean relative spectral power between individuals with and without insomnia.

### 3.4 Comparison of WESI in individuals with and without insomnia

**FIGURE 4:**
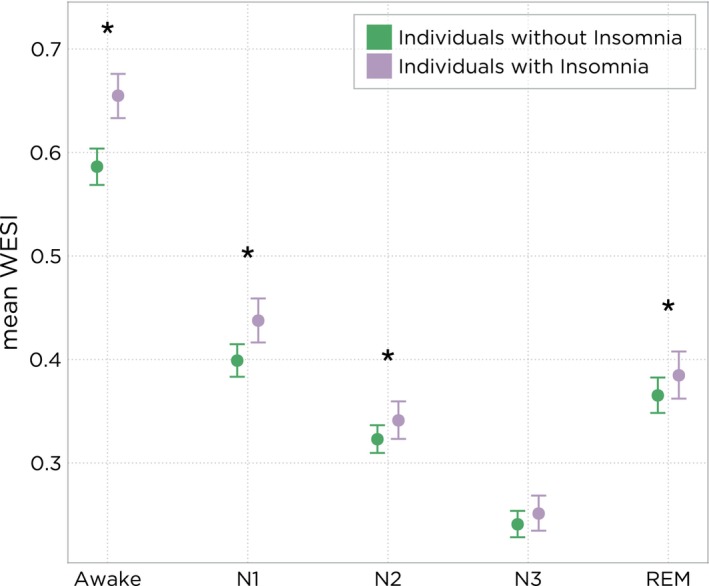
Mean (95% confidence interval [CI]) of the Wake Electroencephalographic Similarity Index (WESI) feature in individuals with and without insomnia. The *y*‐axis indicates the WESI score for the given sleep stage. WESI was trained with 3‐s spectral patterns of awake periods labelled as 1 and sleep periods labelled as 0. WESI scores closer to 1 denote a segment was more like awake spectral patterns, and WESI scores closer to 0 denote a segment was more like sleep spectral patterns. *Indicates statistically significant difference (*p* < 0.05) between groups (with minus without insomnia).

Higher WESI scores indicate more wake‐like EEG patterns regardless of scored sleep stage (Figure 4). During Wake, individuals with insomnia had higher WESI scores, with a mean of 0.654 (95% CI: 0.633, 0.675) compared with 0.586 (95% CI: 0.568, 0.603) in non‐insomnia individuals, with a difference between these two groups of 0.068 (95% CI: 0.054, 0.083, *p* < 0.001). Individuals with insomnia showed higher WESI scores in N1 sleep (0.039 points higher; 95% CI: 0.025, 0.053; *p* < 0.001), N2 sleep (0.018 points higher, 95% CI: 0.007, 0.030; *p* = 0.003) and REM sleep (0.020 points higher, 95% CI: 0.005, 0.034; *p* = 0.004). There was no statistically significant difference in N3 between the two groups.

### 3.5 Comparison of spindle features in individuals with and without insomnia

**FIGURE 5:**
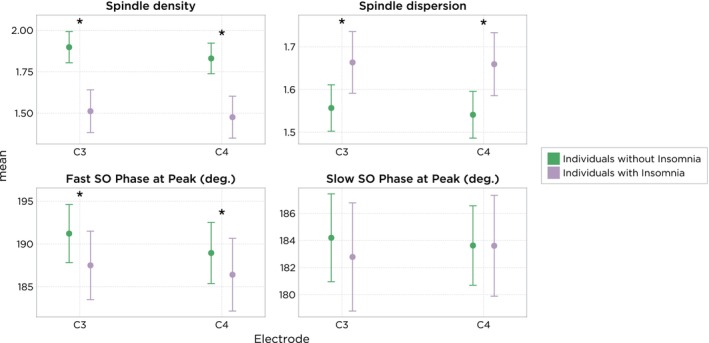
Spindle features in individuals with and without insomnia (mean, 95% confidence interval [CI]). SO, slow oscillations. *Indicates statistically significant difference (*p* < 0.05) between groups (with minus without insomnia). Frequency range for fast (≥ 13–15 Hz) and slow (11–13 Hz) spindles.

Spindle density (number of sleep spindles per minute) was reduced in individuals with insomnia when compared with controls, ranging from −0.35 (C4 electrode: 95% CI: −0.44, −0.27; *p* < 0.001) to −0.39 (C3 electrode: 95% CI: −0.47, −0.30; *p* < 0.001), and dispersion (a marker for the spread of the spindles) was increased at both central recording sites by 0.11 (C3 electrode: 95% CI: 0.06, 0.15; *p* < 0.001) and 0.12 (C4 electrode: 95% CI: 0.07, 0.17; *p* < 0.001) in individuals with insomnia when compared with non‐insomnia individuals (Figure 5). The SO phase at the peak of fast spindles was decreased in individuals with insomnia when compared with non‐insomnia individuals. The decrease in phase observed in individuals with insomnia ranged from −2.54 degrees (C4 electrode: 95% CI: −4.85, −0.23; *p* = 0.018) to −3.71 degrees (C3 electrode: 95% CI: −5.82, −1.60; *p* < 0.001).

## 4 DISCUSSION

In an analysis of one of the largest insomnia datasets to date, we found that individuals with insomnia, when compared with individuals without insomnia, have more frequent transitions to Wake from any stage (especially from Wake and N1) and fewer transitions from Wake to all sleep stages. These results demonstrate a drive to wakefulness in individuals with insomnia. In addition, Wake and N1 had more relative alpha power and less relative delta power, suggesting hyperarousal in insomnia subjects. These findings are further supported by higher WESI scores, which are indicative of a more wake‐like EEG pattern in individuals with insomnia. Finally, spindle density was reduced in individuals with insomnia compared with controls, potentially suggesting an instability in sleep‐sustaining microarchitecture.

Several hypotheses on the pathophysiology of chronic insomnia disorder have been proposed, with most evidence to date supporting hyperarousal (Riemann et al., 2015; Van Someren, 2021). We identified reduced transitions to REM from any sleep stage and increased transitions from REM‐to‐Wake in the insomnia group compared with controls. These observations suggest REM fragmentation (Conte et al., 2023; Riemann et al., 2012), which may be influenced by various elements. Notably, the wake‐promoting components of the ascending arousal system, primarily via the locus coeruleus as the main source of noradrenaline, might be hyperactive during REM sleep in insomnia and thus lead to hyperarousal and increased sleep instability (Feige et al., 2023). However, in our analysis the probability of staying in REM sleep did not differ between the two groups, therefore other systems might be involved that may play a significant role in REM sleep regulation in insomnia pathology.

Another hypothesis is that the sleep–wake circuit is disturbed in insomnia (Palagini et al., 2023). γ‐Aminobutyric acid (GABA)ergic neurons of the ventrolateral preoptic nucleus (VLPO) are a key inhibitor of the ascending arousal system, whereas orexin neurons in the lateral hypothalamus activate this system (Saper et al., 2001). In animals, lesions of the VLPO can produce an insomnia‐like phenotype (Lu et al., 2000), while loss of the orexin neurons in narcolepsy causes sleepiness (De Luca et al., 2022; Lecea & Huerta, 2014). As sleep and arousal systems reciprocally inhibit one another (Saper et al., 2005), an imbalance might contribute to the sleep issues seen in insomnia, as evidenced by the increased wake transitions in the insomnia group. However, the neurobiological mechanisms underlying these observations in people with insomnia remain poorly understood.

Building on this neurobiological context, the complexity of insomnia's heterogeneity is further elucidated by EEG studies that not only quantify disease severity through parameters such as reduced TST, but also through qualitative changes in brain activity (Andrillon et al., 2020; Zhao et al., 2021). Among these are sleep spindles, which provide further insight into the pathophysiology of this disease given their hypothesized involvement in maintaining sleep stability and memory consolidation (Buysse et al., 2010). Here, we found consistently lower spindle density and higher spindle dispersion in individuals with insomnia. Altered sleep spindle properties have been previously described in individuals with paradoxical insomnia; however, results have not been consistently observed across groups (Andrillon et al., 2020; Bastien et al., 2009; Normand et al., 2016). Additionally, the observed decrease in SO spindle phase peak may suggest that individuals with insomnia have dysregulated thalamocortical activity (Kim et al., 2021), which has been previously linked to sleep misperception (Zou et al., 2021) that may underlie cognitive hyperarousal due to excessive information processing during sleep (Riemann et al., 2010).

Using EEG spectral analysis, we identified further differences in this population as compared with non‐insomnia controls. We found increased alpha and lower delta power in Wake and N1 sleep. As previously noted, it has been hypothesized that patients with insomnia have dysfunction of wake‐ and sleep‐promoting circuits (Scammell et al., 2017), and increased reactivity to internal and external stimuli (Van Someren, 2021). Spectral changes observed in this study support these hypotheses, with evidence of hyperarousal manifesting as increased alpha, a marker of the top‐down regulation of cortical activation (Halgren et al., 2019). This is a possible explanation for why insomnia sufferers have difficulty initiating sleep (Perlis et al., 2022), and experience both reduced quality and intensity once it occurs (Long et al., 2021), further underscored by the decrease in relative delta power during Wake and N1 sleep that might be associated with reduced drowsiness. Notably, we did not observe spectral differences in N3 sleep, suggesting that brain networks involved in the regulation of this sleep stage may not be substantially impaired in individuals with insomnia (Van Someren, 2021; Wei et al., 2017), although the overall amount of N3 sleep is reduced in insomnia (Baglioni et al., 2014). Interestingly, we found no increase in beta activity, a marker of cognitive processing, in individuals with insomnia, different from what has been reported in prior studies (Shi et al., 2022; Zhao et al., 2021). This discrepancy highlights the heterogeneity of insomnia and may reflect differences in participant selection (e.g. the current study excluded individuals taking benzodiazepines and other hypnotics; Kang et al., 2022), differences in the frequency range used to define beta activity (Zhao et al., 2021), or may be due to not taking into consideration the effect of age and sex in the statistical models, factors that impact EEG activity (Djonlagic et al., 2020; Svetnik et al., 2017).

To delve further into the pathophysiology of insomnia, we developed a machine learning technique, the WESI. This multidimensional analytical approach approximates sleep depth, using a combination of spectral features. Here, we observed that individuals with insomnia had consistently higher scores across all stages except for N3, indicating lower sleep depth and more wake‐like characteristics across both NREM and REM sleep. Employing this model helped identify differences in individuals with insomnia where individual EEG features failed to do so, supporting the complexity and heterogeneity of the condition. Based on these results, we conclude that individuals with insomnia have difficulty fully achieving the sleep depth of controls, possibly due to a persistent state of hyperarousal (Christensen et al., 2019).

Despite utilizing one of the largest insomnia groups reported, the current study has limitations that should be considered when interpreting these results. First, pooling individuals from different recording sites and datasets, along with the inclusion of diverse insomnia groups, such as combining DSM‐5 diagnosed insomnia disorder, with non‐DMS‐5 insomnia, introduces confounders that could reduce differences and thus obscure otherwise significant findings. It is important to consider that acute and chronic manifestations of the disease can present different pathophysiological markers. For example, acute forms of insomnia could be characterized by a significant increase in sympathetic activation, but these differences might diminish when the condition becomes chronic leading to the inability to inhibit excessive wakefulness (Vargas et al., 2020). Nevertheless, this was partially mitigated by using objective inclusion criteria to define a relatively homogenous population, and by accounting for age, sex and recoding variability in the statistical analysis that should improve the generalizability of the results. A further limitation pertains to the use of individuals without insomnia from the Beacon and SHHS datasets. As the Idorsia dataset is derived from two registered Phase 3 clinical trials, it contains only patients diagnosed with chronic insomnia, without direct age‐matched controls without insomnia. Thus, this discrepancy could affect the strength of comparative analyses between insomnia and non‐insomnia individuals further driven by a “first‐night effect” that might be different between groups. Third, the selected cut‐off for insomnia could restrict the applicability of our findings to individuals with insomnia with objective short sleep and not to individuals with insomnia but without short sleep durations. Furthermore, the changes in relative power reported here leave out a more granular understanding of spectral differences: for instance, as we examined only relative power, changes in delta, which tends to include more power overall, may have influenced the power of other spectral bands. Likewise, because we did not isolate the aperiodic power spectra, it is not clear to what extent the differences we identified were driven by periodic versus aperiodic components of the underlying EEG signal. Finally, we did not assess differences in individual NREM–REM sleep cycles, instead averaging across the entire night and therefore possibly missing cycle‐dependent changes in the dynamics of sleep architecture.

## 5 CONCLUSION

Our findings confirm that insomnia is likely due to a dysfunction both in sleep quantity and quality, as well as to hyperarousal. Assessing the impact of insomnia therapies on these sleep measurements will be of interest for future studies.

## AUTHOR CONTRIBUTIONS

**Tobias Di Marco:** Methodology; writing – review and editing; writing – original draft; formal analysis; conceptualization; visualization; project administration; data curation; supervision. **Thomas E. Scammell:** Writing – review and editing; conceptualization; investigation; methodology; supervision. **Kolia Sadeghi:** Software; formal analysis; methodology; visualization; data curation; supervision; writing – review and editing; validation. **Alexandre N. Datta:** Methodology; writing – review and editing; supervision; visualization; conceptualization. **David Little:** Writing – review and editing; methodology; data curation; visualization; software; formal analysis; supervision; validation. **Nurkurniati Tjiptarto:** Methodology; visualization; writing – review and editing; software; formal analysis; data curation; supervision; validation. **Ina Djonlagic:** Conceptualization; investigation; writing – review and editing; methodology; supervision. **Antonio Olivieri:** Writing – review and editing; funding acquisition; resources; conceptualization; methodology; supervision. **Gary Zammit:** Writing – review and editing; methodology; conceptualization; supervision. **Andrew Krystal:** Writing – review and editing; supervision; conceptualization; methodology. **Jay Pathmanathan:** Writing – review and editing; methodology; validation; visualization; software; formal analysis; data curation; supervision; conceptualization. **Jacob Donoghue:** Conceptualization; methodology; validation; visualization; writing – review and editing; software; formal analysis; data curation; supervision. **Jeffrey Hubbard:** Writing – review and editing; conceptualization; investigation; methodology; visualization; project administration; supervision. **Yves Dauvilliers:** Conceptualization; investigation; writing – review and editing; methodology; supervision; visualization.

## FUNDING INFORMATION

The study was supported by Idorsia Pharmaceuticals Ltd.

## CONFLICT OF INTEREST STATEMENT

Y.D. reports board membership, consultancy and lecture activity with Idorsia. A.N.D. reports consultancy, and lecture activity with Idorsia, Neurocrine, Epilog, Roche and Jazz Pharmaceuticals. A.K. reports research grants from Janssen Pharmaceuticals, Axsome Pharmaceutics, Attune, Harmony, Neurocrine Biosciences, Reveal Biosensors, The Ray and Dagmar Dolby Family Fund, and the National Institutes of Health; Consultancy with Axsome Therapeutics, Big Health, Eisai, Evecxia, Harmony Biosciences, Idorsia, Janssen Pharmaceuticals, Jazz Pharmaceuticals, Millenium Pharmaceuticals, Merck, Neurocrine Biosciences, Neurawell, Pernix, Otsuka Pharmaceuticals, Sage, Takeda; and owning stock options for Neurawell, Big‐Health. I.D. reports consultancy and lecture activity with Idorsia. T.E.S. has consulted for Avadel, Harmony Biosciences, Jazz Pharmaceuticals, and Takeda, and he has received research grants from Jazz Pharmaceuticals, Harmony Biosciences, and Takeda. G.Z. is an employee of Clinilabs Drug Development Corporation, a company that has received grants from Idorsia and reports consultancy activity with Idorsia. D.L., J.D., J.P., K.S. and N.T. are employees of Beacon Biosignals; Beacon Biosignals was funded by Idorsia to conduct the analyses. T.D.M., A.O. and J.H. are employees of Idorsia. Non‐financial disclosure: none.

## Supporting information


**DATA S1** Supporting Information.

**DATA S2** Supporting Information.

## Data Availability

In addition to Idorsia's existing clinical trial disclosure activities, the company is committed to implementing the Principles for Responsible Clinical Trial Data Sharing jointly issued by the European Federation of Pharmaceutical Industries and Associations (EFPIA) and the Pharmaceutical Research and Manufacturers of America (PhRMA). Requests for data sharing, of any level, can be directed to clinical–trials–disclosure@idorsia.com for medical and scientific evaluation.
