# Supplementary material for: Hyperarousal features in the sleep architecture of individuals with and without insomnia
Source: J Sleep Res. 2024 Jun 9;34(1):e14256. doi: 10.1111/jsr.14256 (PMC11744246; doi:10.1111/jsr.14256)
Supplement: Supplementary file 1 — DATA S1 Supporting Information. [file JSR-34-e14256-s002.docx]

**Sleep architecture in individuals with and without insomnia**

Tobias Di Marco^1,2^, Thomas E. Scammell^3,^ Kolia Sadeghi^4^, Alexandre N. Datta^5^, David Little^4^, Nurkurniati Tjiptarto^4^, Ina Djonlagic^3^, Antonio Olivieri^1^, Gary Zammit^6^, Andrew Krystal^7^, Jay Pathmanathan^4^, Jacob Donoghue^4^, Jeffrey Hubbard^1^, Yves Dauvilliers^8^

1. Idorsia Pharmaceuticals Ltd, 4123, Allschwil, Switzerland

2. Department of Clinical Research, University of Basel, Schanzenstrasse 55, 4031 Basel, Switzerland

3. Department of Neurology, Beth Israel Deaconess Medical Center, Boston, MA, United States

4. Beacon Biosignals, Inc., Boston, MA, United States

5. University Children’s Hospital Basel, Basel, Switzerland

6. Clinilabs Drug Development Corporation, New York, United States

7. University of California, San Francisco, CA, USA

8. Centre National de Référence Narcolepsie, Unité du Sommeil, CHU Montpellier, Hôpital Gui–de–Chauliac, Université de Montpellier, INSERM INM, Montpellier, France

**Corresponding author:**

1. Tobias Di Marco

Idorsia Pharmaceuticals Ltd, Hegenheimermattweg 91, 4123 Allschwil, Switzerland.

E–mail: t[obias.dimarco@unibas.ch](mailto:Tobias.dimarco@unibas.ch)

1. Yves Dauvilliers

Université de Montpellier, Montpellier, France

E-mail: y-dauvilliers@chu-montpellier.fr

**Target journal:** *SLEEP*

# Supplement

| **ICD 10 Diagnosis** | | | |
| --- | --- | --- | --- |
| F24 | psychotic disorder | G47.52 | REM sleep behavior disorder |
| F33 | major depressive disorder | G47.26 | Circadian rhythm sleep disorder, shift work type |
| S09.90 | traumatic brain injury | G47.14 | Hypersomnia due to medical condition |
| F20 | schizophrenia | G47.32 | High altitude periodic breathing |
| G30 | alzheimer's disease | G47.421 | Narcolepsy in conditions classified elsewhere with cataplexy |
| G40 | epilepsy | G47.22 | Circadian rhythm sleep disorder, advanced sleep phase type |
| S07.1 | traumatic brain injury | G47.50 | Parasomnia, unspecified |
| S02.1 | traumatic brain injury | G47.30 | Sleep apnea, unspecified |
| I63 | arterial ischemic stroke | G47.429 | Narcolepsy in conditions classified elsewhere without cataplexy |
| G93 | anoxic encephalopathy | G47.33 | Obstructive sleep apnea (adult) (pediatric) |
| Z86.73 | history of ischemic left ica stroke | G47.36 | Sleep related hypoventilation in conditions classified elsewhere |
| F80 | developmental delay | G47.9 | Sleep disorder, unspecified |
| G23 | progressive supranuclear ophthalmoplegia | G47.34 | Idiopathic sleep related nonobstructive alveolar hypoventilation |
| F02 | dementia in other diseases classified elsewhere | G47.63 | Sleep related bruxism |
| G20 | parkinson's disease | G47.19 | Other hypersomnia |
| G31.84 | cognitive impairment | G47.69 | Other sleep related movement disorders |
| F25 | schizoaffective disorder | G47.39 | Other sleep apnea |
| F05 | delirium | G47.10 | Hypersomnia, unspecified |
| G80 | cerebral palsy | G47.35 | Congenital central alveolar hypoventilation syndrome |
| F31 | bipolar 1 disorder | G47.27 | Circadian rhythm sleep disorder in conditions classified elsewhere |
| S02.0 | traumatic brain injury | G47.31 | Primary central sleep apnea |
| G35 | multiple sclerosis | G47.20 | Circadian rhythm sleep disorder, unspecified type |
| S06 | stroke, hemorrhagic | G47.37 | Central sleep apnea in conditions classified elsewhere |
| G91 | hydrocephalus | G47.419 | Narcolepsy without cataplexy |
| G47.61 | Periodic limb movement disorder | G47.8 | Other sleep disorders |
| G47.53 | Recurrent isolated sleep paralysis | G47.411 | Narcolepsy with cataplexy |
| G47.13 | Recurrent hypersomnia | G47.23 | Circadian rhythm sleep disorder, irregular sleep wake type |
| G47.12 | Idiopathic hypersomnia without long sleep time | G47.54 | Parasomnia in conditions classified elsewhere |
| G47.25 | Circadian rhythm sleep disorder, jet lag type | G47.21 | Circadian rhythm sleep disorder, delayed sleep phase type |
| G47.59 | Other parasomnia | G47.11 | Idiopathic hypersomnia with long sleep time |
| G47.24 | Circadian rhythm sleep disorder, free running type | G47.29 | Other circadian rhythm sleep disorder |
| G47.62 | Sleep related leg cramps | G47.51 | Confusional arousals |

Supplement 1: ICD–10 criteria for exclusion for the Beacon dataset.

| **Drug class** | **Medications** |
| --- | --- |
| **Sedating Antihistamines** | |
|  | triprolidine |
|  | acrivastine |
|  | chlorpheniramine |
|  | doxylamine |
|  | ketotifen |
|  | promethazine |
|  | diphenhydramine |
|  | dimenhydrinate |
|  | azatadine |
|  | timeprazine |
| **Centrally acting anticholinergics** | |
|  | carbinoxamine |
|  | oxybutynin |
|  | solifenacin |
|  | tropatepine |
| **Stimulants** | |
|  | amphetamine |
|  | ephedrine |
|  | modafinil |
|  | armodafinil |
|  | methylphenidate |
| **Antidepressants** | |
|  | doxepin |
|  | trazodone or desyrel |
|  | bupropion |
|  | citalopram |
|  | duloxetine |
|  | escitalopram |
|  | fluoxetine |
|  | paroxetine |
|  | sertraline |
|  | amitriptyline |
|  | trimipramine |
|  | venlafaxine |
|  | selegiline |
|  | lithium |
|  | fluvoxamine |
|  | moclobemide |
|  | nefazodone |
| **Antipsychotics** | |
|  | quetiapine |
|  | aripiprazole |
|  | olanzapine |
| **Anxiolytics** | |
|  | alprazolam |
|  | chlordiazepoxide or librium |
|  | clorazepate |
|  | diazepam |
|  | lorazepam |
|  | midazolam or versed |
|  | oxazepam or serax |
|  | hydroxyzine |
|  | clomipramine |
|  | desipramine |
|  | imipramine |
|  | mirtazapine |
|  | nortriptyline |
|  | buspirone |
| **Hypnotics** | |
|  | zolpidem |
|  | estazolam |
|  | prosom |
|  | flurazepam |
|  | temazepam |
|  | triazolam |
|  | quazepam |
|  | suvorexant |
|  | butisol |
|  | butabarbital |
|  | tasimelteon or hetlioz |
|  | eszopiclone or lunesta |
|  | ramelteon |
|  | zaleplon or sonata |
|  | tryptophan |
|  | melatonin |
| **Cholinesterase inhibitors** | |
|  | donepezil |
|  | galantamine |
| **Mood stabilizers or anticonvulsants** | |
|  | clonazepam or klonopin |
|  | clobazam or onfi |
|  | secobarbital or seconal |
|  | carbamazepine |
|  | gabapentin |
|  | lamotrigine |
|  | oxcarbazepine |
|  | pregabalin |
|  | tiagabine |
|  | phenytoin |
|  | ethotoin |
|  | fosphenytoin |
|  | primidone |
|  | vigabatrin |
|  | ethosuximide |
|  | mephenytoin |
|  | valproic acid |
|  | mephobarbital |
| **Other** | |
|  | pramipexole |
|  | levodopa |
|  | dextromethorphan |
|  | ticlopidine |
|  | dexamethasone |
|  | prednisone |
|  | isotrenitoin |
|  | methylprednisone |
|  | hydracarbazine |
|  | heparin |

Supplement 2: Medications leading to the exclusion from the insomnia and non–insomnia group in the Beacon dataset.


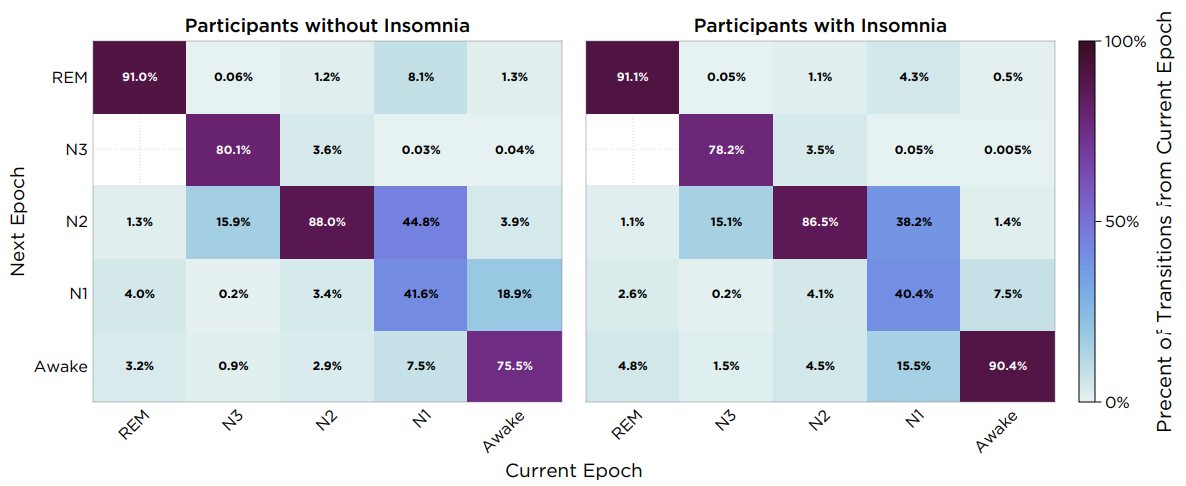


Supplement 3 Transition matrix illustrating the model-estimated probabilities of sleep–wake transitions. The color gradient represents the absolute percentage in transitions for each group (individuals without and with insomnia).
