# Supplementary figures and images for: Hyperarousal features in the sleep architecture of individuals with and without insomnia
Source: J Sleep Res. 2024 Jun 9;34(1):e14256. doi: 10.1111/jsr.14256 (PMC11744246; doi:10.1111/jsr.14256)

Individuals without Insomnia

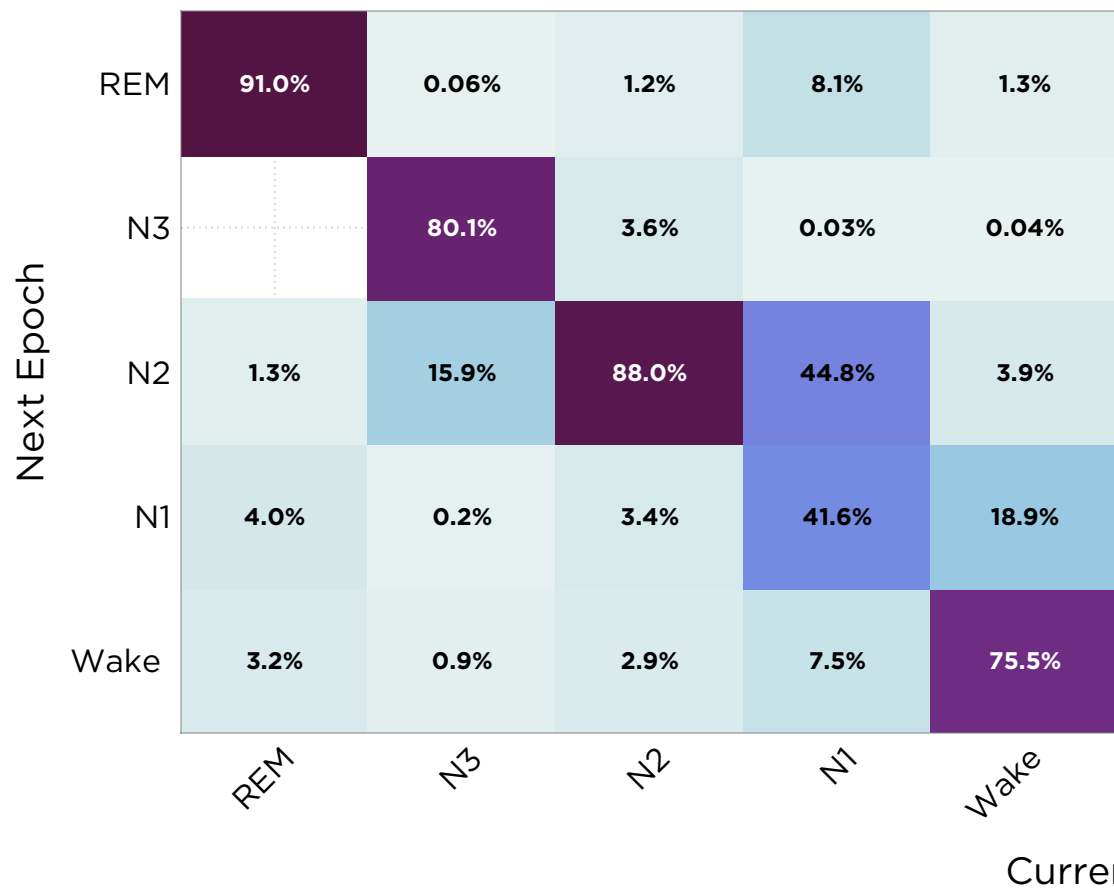

Individuals with Insomnia

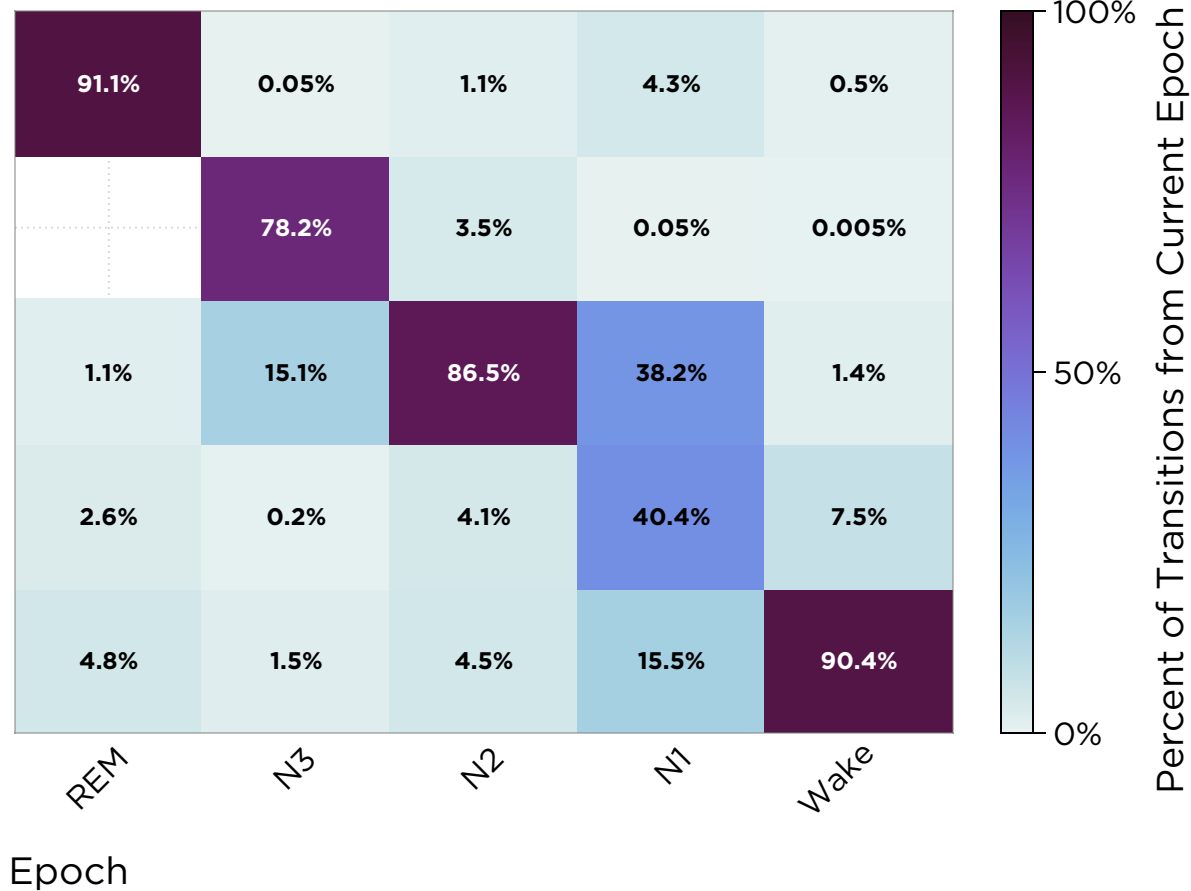

Supplement: Supplementary file 2 — DATA S2 Supporting Information. [file JSR-34-e14256-s001.pdf]
